# In Silico Identification of Candidate Genes for Fertility Restoration in Cytoplasmic Male Sterile Perennial Ryegrass (*Lolium perenne* L.)

**DOI:** 10.1093/gbe/evw047

**Published:** 2016-03-04

**Authors:** Timothy Sykes, Steven Yates, Istvan Nagy, Torben Asp, Ian Small, Bruno Studer

**Affiliations:** 1 Institute of Agricultural Sciences, Forage Crop Genetics, ETH Zurich, Zurich, Switzerland; 2 Department of Molecular Biology and Genetics, Research Centre Flakkebjerg, Aarhus University, Slagelse, Denmark; 3 Plant Energy Biology, ARC Centre of Excellence, the University of Western Australia, Crawley, Western Australia, Australia

**Keywords:** cytoplasmic male sterility (CMS), hybrid breeding, pentatricopeptide repeat (PPR) proteins, perennial ryegrass (Lolium perenne L.), restoration of fertility, restorer of fertility-like PPR (RFL)

## Abstract

Perennial ryegrass (*Lolium perenne* L.) is widely used for forage production in both permanent and temporary grassland systems. To increase yields in perennial ryegrass, recent breeding efforts have been focused on strategies to more efficiently exploit heterosis by hybrid breeding. Cytoplasmic male sterility (CMS) is a widely applied mechanism to control pollination for commercial hybrid seed production and although CMS systems have been identified in perennial ryegrass, they are yet to be fully characterized. Here, we present a bioinformatics pipeline for efficient identification of candidate restorer of fertility (*Rf*) genes for CMS. From a high-quality draft of the perennial ryegrass genome, 373 *pentatricopeptide repeat (PPR)* genes were identified and classified, further identifying 25 *restorer of fertility-like PPR* (*RFL*) genes through a combination of DNA sequence clustering and comparison to known *Rf* genes. This extensive gene family was targeted as the majority of *Rf* genes in higher plants are *RFL* genes. These *RFL* genes were further investigated by phylogenetic analyses, identifying three groups of perennial ryegrass *RFL*s. These three groups likely represent genomic regions of active *RFL* generation and identify the probable location of perennial ryegrass *PPR-Rf* genes. This pipeline allows for the identification of candidate *PPR-Rf* genes from genomic sequence data and can be used in any plant species. Functional markers for *PPR-Rf* genes will facilitate map-based cloning of *Rf* genes and enable the use of CMS as an efficient tool to control pollination for hybrid crop production.

## Introduction

The agronomical value of perennial ryegrass (*Lolium perenne* L.) comes from its ability to produce high forage yield of good feed quality in both permanent and temporary grassland systems (Wilkins 1991). Due to the increasing global demand for animal products, improved varieties of forage grasses are becoming an important aspect of global food security. Thus, perennial ryegrass has been the subject of intensive breeding efforts over recent decades. However, these breeding efforts are mainly focused on the improvement of population and synthetic varieties and show limited increases in biomass yield (van der Heijden and Roulund 2010; Pembleton et al. 2015), which is one of the most important traits in forage grasses.

Hybrid breeding, by efficiently exploiting the phenomenon of heterosis, has been successfully used in breeding programs to increase yield in several important crop species including rice (*Oryza sativa* L.), maize (*Zea mays* L.), and rapeseed (*Brassica napus* L.) (Duvick 2001; Melchinger 2010). Due to its significant impact, there are currently considerable efforts to establish hybrid breeding schemes for other crops including wheat (*Triticum aestivium* L.) (Longin et al. 2012). The development and application of hybrid breeding in forage crops has the potential to result in similar yield increases (Pembleton et al. 2015). To employ hybrid breeding in perennial ryegrass, one of the major challenges is the absence of a pollination control strategy that would allow the efficient production of hybrid seed on a commercial level. In several plant species including maize, onion (*Allium cepa* L.), sorghum (*Sorghum bicolor* L.), sugar beet (*Beta vulgaris* L.), sunflower (*Helianthus annuus* L.), rapeseed, common beans (*Phaseolus vulgaris* L.), and rice, cytoplasmic male sterility (CMS) has been successfully applied to control pollination for hybrid seed production (Ahokas 1983; Yuan and Virmani 1988; Virmani 1994; Schnable and Wise 1998; Havey 2004; Martin et al. 2009; Singh et al. 2010; Kubo et al. 2011). Although CMS systems have been identified in perennial ryegrass (Wit 1974; Connoly and Wright-Turner 1984; Creemersmolenaar et al. 1992) , they are yet to be fully characterized (Kiang et al. 1993; Kiang and Kavanagh 1996; McDermott et al. 2008; Islam et al. 2014).

CMS in flowering plants is characterized by a maternally inherited inability to produce functional pollen (Hanson and Bentolila 2004). This functional defect is often attributed to aberrant transcripts originating from the mitochondrial genome, with these CMS causing transcripts usually coding for novel chimeric open reading frames (ORFs) containing part of a functional mitochondrial gene (Chase and Babay-Laughnan 2004; Hanson and Bentolila 2004). The translated products of these chimeric transcripts disrupt normal mitochondrial function such that the energy requirements for pollen formation cannot be met, rendering the pollen unviable (Schnable and Wise 1998).

The CMS phenotype is often restored through the action of nuclear-derived RNA-binding proteins that are generally members of the large family of pentatricopeptide repeat (PPR) proteins (Barkan and Small 2014). Exceptions are the CMS-T restoration in maize (Cui et al. 1996), the *restorer of fertility* (*Rf*) gene *bvORF20* in sugar beet (Kitazaki et al. 2015) as well as other RNA-binding proteins that have been implicated in fertility restoration (Itabashi et al. 2011; Hu et al. 2012). PPR proteins are particularly numerous in land plants, with 450 PPRs identified in Arabidopsis (*Arabidopsis thaliana* L.) and 477 in rice (Schmitz-Linneweber and Small 2008; Zehrmann et al. 2009; Chateigner-Boutin and Small 2010; Castandet and Araya 2011; Fujii and Small 2011). Although PPR proteins are encoded by the nuclear genome, they most often function within organelles to mediate gene expression, facilitating the processing, and translation of RNAs (Small et al. 2013). PPR proteins contain tandem arrays of a degenerate 35 amino acid motif that bind to RNA in a sequence-specific manner (Schmitz-Linneweber and Small 2008). PPR proteins appear to be functional only in organelles and as such have been described as the chaperones of organelle gene expression (Colcombet et al. 2013). PPR proteins have previously been divided into subclasses based on PPR motif variations and a series of conserved C-terminal domains (Lurin et al. 2004; O'Toole et al. 2008). The two main subclasses of PPR proteins, the P and PLS subclasses, are defined by the organization of the individual PPR motifs within a PPR gene. The P-type PPRs are comprised almost entirely from the canonical 35-amino acid P motif. In contrast, the PLS subclass of PPRs is composed of triplet repeats containing one P motif, one L motif (“long,” usually 36 amino acids) and one S (“short,” usually 31 amino acids). This PLS subclass is also characterized by three distinctive C-terminal motifs; E (extended), E+ (slightly longer version of the E-domain), and DYW (named for terminating with a conserved Asp-Tyr-Trp triplet). All PPRs that have been shown to be involved in RNA editing, in both mitochondria and chloroplasts, are members of these three subgroups (Schallenberg-Ruedinger et al. 2013). The E/E+ domains are believed to provide an essential recognition site for an (as yet unidentified) editing complex. The DYW domain, which usually includes an E domain, shows similarity to deaminases and is possibly directly involved in RNA editing (Hammani et al. 2009; Okuda et al. 2009; Tasaki et al. 2010; Okuda and Shikanai 2012; Toda et al. 2012).

A subgroup of the P-type PPRs is specifically linked to fertility restoration of CMS; the restorer of fertility-like PPR (RFL) proteins. This group is identified by their relative homology from within the PPR family, their identity with other known CMS restorer PPRs from related plant species and their tendency to be present in several homologous copies clustered within the genome. These RFLs comprise around 10–30 members per plant genome from the full set of PPRs (Andres et al. 2007; Fujii et al. 2011). It has been shown previously that *RFL* genes appear to be under different selection pressures when compared with the rest of the *PPR* gene family members. Within the *RFL* subgroup, high ratio of nonsynonymous versus synonymous nucleotide substitutions indicates diversifying selection (Geddy and Brown 2007; Fujii et al. 2011). This suggests, in conjunction with gene duplication, that the generation of new *RFL* genes and subsequent loss of nonfunctional *RFL*s is relatively rapid, keeping pace with the generation of novel CMS sources. CMS is also used as a model system for studying nuclear/mitochondrial genome interactions, as its easy detection allows researchers to rapidly identify individuals with a breakdown in nuclear/mitochondrial signaling (Chase 2007).

In order to provide plant breeders with a molecular tool for candidate *Rf* gene identification and thus facilitate the implementation of hybrid breeding schemes in perennial ryegrass, this study aimed to locate, in silico, regions of active *RFL* generation in the perennial ryegrass genome by 1) the development and validation of a bioinformatics pipeline for the identification of *PPR*s and *RFL*s from genomic sequence, 2) utilizing this pipeline for identification of *PPR* genes within the perennial ryegrass genome, 3) classifying these *PPR* genes in order to isolate the *RFL*s as potential candidate *Rf* genes, 4) phylogenetically analyzing the *RFL* genes from several grass species to identify groups of rapidly diverging *RFL* genes within the perennial ryegrass genome, and 5) using this analysis to locate genomic regions of novel *RFL* generation.

## Materials and Methods

### Identification of PPR Proteins

To identify, in silico, members of the PPR protein family in the genome assembly of perennial ryegrass (http://185.45.23.197:5080/ryegrassgenome), all available PPR domain sequences from the Pfam database (http://pfam.xfam.org) were collected and used for the development of a Hidden Markov Model (HMM) profile matrix using the *hmmbuild* program of the HMMER package (v3.1b1, http://hmmer.org). This HMM profile matrix was used to identify members of the PPR family in a total of 71,009 translated DNA transcript sequences obtained from ab initio and evidence-based predictions from a high-quality genomic draft of the perennial ryegrass genome sequence (Byrne et al. 2015).

### Classification of PPR Proteins

PPR-containing transcript sequences were analyzed on a standalone PfamScan pipeline to ascertain the exact co-ordinates of each PPR domain within a scaffold sequence as well as information on the frequencies and distribution of the PPR domains. Predictive information on protein functions and conserved sequence elements was obtained by sending all PPR containing sequences through a standalone InterProScan (version 5; Jones et al. 2014) pipeline by scanning the PANTHER, PROSITE profiles, Pfam, and SUPERFAMILY databases. Sequences were identified as belonging to the P or PLS subfamilies through analysis of PPR motif lengths, with the PLS subfamily having longer (L) and shorter (S) subdomains (Lurin et al. 2004). The identified members of the PLS family were processed using the online domain elicitation tool MEME (Bailey and Gribskov 1998) and conserved blocks representing the E, E + ,  and DYW C-terminal domains identified. To ensure all possible C-terminal domains have been identified, the PPR domains were masked out using the *maskfeat* program of the EMBOSS package (Rice et al. 2000). The masked sequences were aligned and clustered to identify any conserved regions outside of the PPR domains. All sequences were also searched using HMM profiles for the E, E + , and DYW domains.

### Identification of RFL Proteins

All identified PPR genomic sequences were clustered using CD-hit (Li and Godzik 2006) at 90%, 80%, 70%, 60%, and 50% identity. Clustering at 90–70% revealed no clusters of more than three members. Clustering at 60% revealed three clusters containing 9, 6, and 4 PPRs, respectively. All PPR sequences were then aligned, using the NCBI BLAST platform (http://blast.ncbi.nlm.nih.gov/), to known or predicted restorer genes from; brachypodium (gi|357139997), rice (gi|33859441), and maize (gi|662249846). Hits with at least 50% identity and 50% query cover were collected. PPRs that were present on at least three of these four lists were considered candidate RFLs.

### Databases

The coding sequences (CDS) of the following species were downloaded from Ensembl Plants (http://plants.ensembl.org/index.html, on April 10, 2014) (Flicek et al. 2012) using the Perl API tool (McLaren et al. 2010); *A. thaliana* (TAIR10), *Brachypodium distachyon* (V1.0), *Hordeum vulgare* (European Nucleotide assembly (ENA): GCA_000326085.1), *Musa acuminata* (ENA: GCA_000313855.1), *O. sativa Japonica* (ENA: GCA_000005425.2), *Setaria italica* (ENA: GCA_000263155.1), *S. bicolor* (ENA: GCA_000003195.1), *Triticum urartu* (ENA: GCA_000347455.1), *Z. mays* (ENA: GCA_000005005.5). The following CDS of *Phyllostachys heterocla* da (v1.0) was downloaded from http://www.bamboogdb.rg/ (Peng et al. 2013). The CDS of *L. perenne* was received from Ruttink et al. (2013) and the respective CDS of *Lolium multiflorum* and *Festuca pratense* were kindly provided by Stoces et al. (in preparation). The *Eragrostis tef* cDNA was downloaded from http://www.tef-research.org/genome.html (Extended.gte200.cDNA.fa, Cannarozzi et al. 2014) and its CDS determined using ORFprdictor (Min et al. 2005). The cDNA was then searched against a protein BLAST database comprising of *A. thaliana*, *Glycine max*, *O. sativa Japonica*, *Populus trichocarpa*, and *Manihot esculenta*, using BLASTP (Altschul et al. 1990) with minimum e-value 1e ^−^ ^5^. The BLASTP results were used to infer coding frame, all other parameters and methods used were as described by Min et al. (2005).

### Orthologous Clustering of Species

To cluster the protein sequences into orthologous clusters, the offline version of OrthoMCL (Li et al. 2003) was used. Briefly, the protein names within a fasta file (per species) were first changed for consistency (also for simplicity) and to ameliorate any problems arising later from special characters and similarities between names. This was done using an in house Perl script. The resulting fasta file was then formatted to make it compliant with the OrthoMCL algorithm (a short species-specific prefix was added to each name for subsequent species identification). The sequences were then filtered for low quality, based on sequence length (>30 aa, retained) and percentage of stop codons (>10%, discarded). From these high quality proteins, an all-versus-all BLASTP was run where all proteins were searched against all proteins (minimum E-value 1e ^−^ ^5^); the database was not split into subgroups when doing this so no corrections for E-score where necessary. The results of the BLASTP were collated and then parsed before loading into a local MySQL orthoMCL database. In the next stage, pairs of proteins that are potentially orthologs, in-paralogs or co-orthologs were identified using the OrthoMCL algorithm (Li et al. 2003), where protein pairwise connections were normalized for ortholog pairs between and within species. The resulting potential pairs were then organized in clusters using the MCL alogirthm (Enright et al. 2002). The results were output and the names were changed back to their original for subsequent work.

### Phylogenetic Reconstruction and Analysis

The phylogenetic relationships between the protein sequences from the OrthoMCL generated cluster containing RFLs, including the nine putative ryegrass RFLs not present, were reconstructed and analyzed using web tools made available by The Montpellier Laboratory of Informatics, Robotics and Microelectronics LIRMM (http://www.phylogeny.fr/; Dereeper et al. 2008). Sequence alignments were completed using MUSCLE (Edgar 2004), phylogenetic analysis using PhyML (Guindon and Gascuel 2003; Anisimova and Gascuel 2006) and the resulting tree viewed using TreeDyn (Chevenet et al. 2006).

## Results

### PPR and RFL Gene Identification and Classification

**Fig. 1.—:**
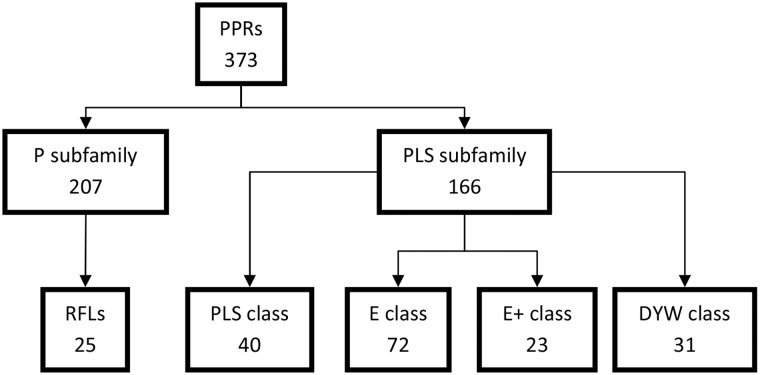
Classification of identified *PPR* genes in perennial ryegrass (*Lolium perenne* L.). Sequences were classified into P and PLS subfamilies, based on the architecture of the repeated PPR motifs. The PLS subfamily was further classified by the presence of several non-PPR C-terminal domains. All identified *RFL* genes were part of the P subfamily.

A draft of the perennial ryegrass genome sequence (Byrne et al. 2015) was scanned to identify *PPR* genes using a HMM profile matrix (Finn et al. 2011). From a total of 71,009 genes, obtained from ab initio and evidence-based gene predictions in the perennial ryegrass genome, 373 *PPR* genes were identified. These 373 *PPR* genes were classified into two subfamilies, P and PLS, based on the arrangements of the repeated PPR motifs. Each of these subfamilies contained roughly half of the identified *PPR* genes with the P subfamily being slightly larger with 207 members, representing 55% of the total *PPR*s. The PLS subfamily was further grouped based on the presence or absence of the C-terminal domains implicated in RNA editing. From a total of 166 PLS subfamily genes, 40 were missing RNA editing-specific C-terminal motifs (PLS subclass), while the remaining 126 were organized into the E class (72), the E+ class (23), and the DYW class (31) (fig. 1). Analysis of the 25 RFLs, identified by homology to known restorers from other grass species, revealed that they all belonged to the P subfamily. Further analysis identified five pseudogenes that were truncated and lacking start/stop codons. These identified *RFLs* have an average of 16 PPR domains as compared with 9.7 PPR domains for the remainder of the *PPR* genes.

### RFL Gene Comparison in Multiple Species

**Fig. 2.—:**
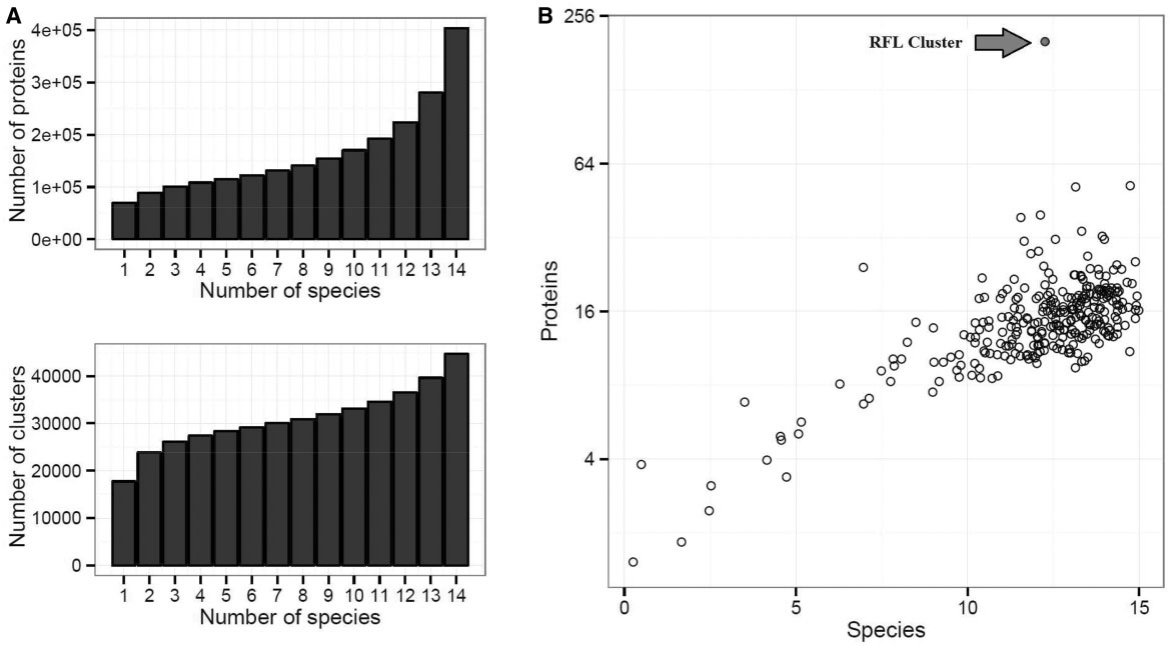
(*A***)** Histogram showing the number of proteins and clusters in relation to the number of species per cluster from the OrthoMCL protein sequence clustering of 14 species. (*B***)** Scatterplot showing the number of species (*x* axis) and the number of proteins (*y* axis, log2 scale) present in the 287 clusters containing at least one perennial ryegrass *PPR* gene. The outlying cluster containing 16 out of 25 identified RFLs is indicated with a red dot.

Orthologous clustering of protein sequences from 14 species was performed to ascertain whether the identified perennial ryegrass *RFL* genes are similar to *RFL* genes from other plant species. For this clustering, the canonical CDS (McLaren et al. 2010) of 14 species were used, comprising a total of 561,090 protein sequences. Of these, 554,468 passed the quality checking by OrthoMCL (Li et al. 2003), of which 403,713 proteins were grouped into 44,672 clusters (fig. 2*A*). A subset of 5,054 clusters contained proteins from all species, representing 30.6% of the 403,713 clustered proteins. In contrast, 17.3% of the sequences were species-specific and contained in 39.7% of clusters (fig. 2*A*).

Further analysis identified 287 clusters that contain at least one of the 373 perennial ryegrass *PPR*s found previously. Plotting the number of species represented in these 287 clusters against the number of proteins present revealed a linear relationship with one clear outlier. This outlying cluster contained 154 proteins originating from 13 species and is more than three times bigger than the second largest cluster. This cluster was entirely composed of PPR proteins and contained 16 of the previously identified 25 RFLs from perennial ryegrass. The nine RFLs not present in this cluster were found to be either pseudogenes or poorly annotated genes leading to them not clustering with the remainder of the RFLs. The following species were dropped: Italian ryegrass (*L. multiflorum* L.) and meadow fescue (*Festuca pratensis* L.) as their sequences originated from transcriptome sequencing and a low number of RFLs were identified; bamboo (*P. heterocla* L.) and teff (*E. tef* L.) as, although their genomes have been sequenced into scaffolds, these were not organized into contiguous sequences and thus did not provide precise information about genome positions. No RFLs were identified from banana (*M. acuminate* L.) This approach not only showed that *RFL* genes form a distinct orthologous group, but also validated the approach used for *RFL* identification within the perennial ryegrass genome.

### Phylogenetic Analysis of the RFL Cluster

**Fig. 3.—:**
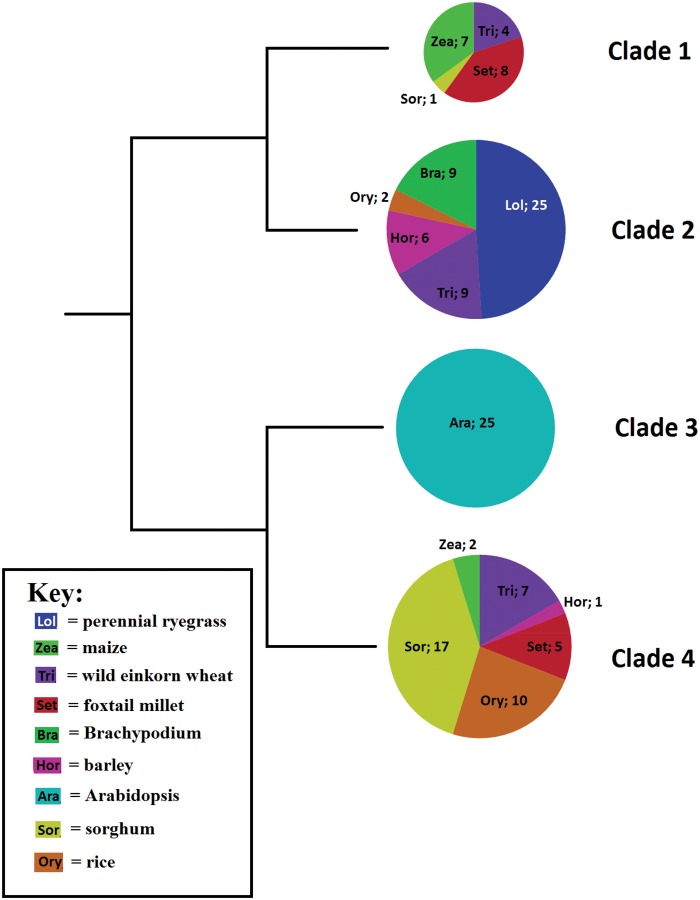
Phylogenetic tree showing the four identified clades within the RFL cluster containing a total of 154 proteins originating from 14 species. Colors are species-specific with abbreviated species names (see table 1) shown along with the number of RFLs present.

Having identified a set of *RFL* genes from multiple species (supplementary table S1, Supplementary Material online), a phylogenetic analysis was performed in order to understand the evolutionary ancestry underpinning the *RFL* genes. Protein sequences from the OrthoMCL generated RFL cluster (red dot in fig. 2*B*) were phylogenetically analyzed, revealing four major clades of RFLs (fig. 3 and table 1). The only dicot included, Arabidopsis, was represented within an entire clade of its own (clade 3). The other three clades encompassed all the monocot sequences with perennial ryegrass and Brachypodium (*B. distachyon* L.) being the only species represented in only one clade and wild einkorn wheat (*T. urartu* L.) being the only species represented in all three monocot clades. All species, with the exceptions of wild einkorn wheat and foxtail millet (*Se. italica* L.), had a majority of sequences present in only one clade.

**Table 1:** The Number of *RFL* Genes in Each Clade as well as the Total Number of *RFL*s Identified Are Given for Each Species

Species	Number of Sequences				Totals
	Clade 1	Clade 2	Clade 3	Clade 4	
*Perennial ryegrass*	—	25	—	—	25
*Wild einkorn Wheat*	4	9	—	7	20
***Barley***	—	6	—	1	7
*Foxtail millet*	8	—	—	5	13
*Rice*	—	2	—	10	12
*Sorghum*	1	—	—	17	18
*Maize*	7	—	—	2	9
*Brachypodium*	—	9	—	—	9
*Arabidopsis*	—	—	28	—	28
Totals	20	51	32	42	145

**Fig. 4.—:**
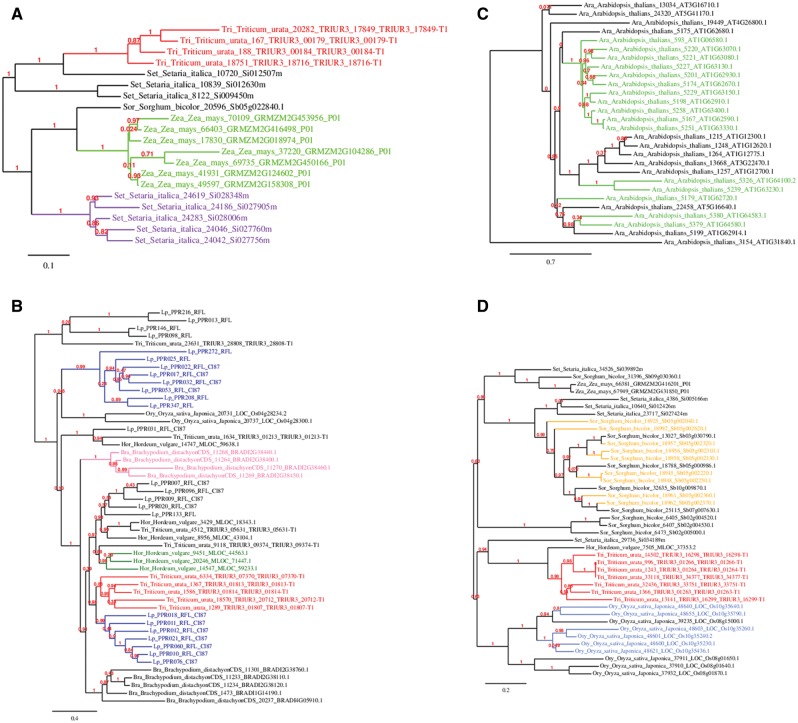
Phylogenetic trees generated using protein sequences from the OrthoMCL generated RFL cluster. (*A*) Clade 1. (*B*) Clade 2. (*C*) Clade 3. (*D*) Clade 4. Colors represent clusters of sequences originating from the same genomic region of that species or showing a similar arrangement when genome information is not available.

To identify the *RFL* genes from each species that most recently evolved, detailed phylogenetic trees of each clade were coupled with genome location data available from Ensemble Plants (http://plants.ensembl.org/index.html). This revealed that within each clade, *RFLs* from the same species tend to cluster together with the tightest clusters containing *RFLs* from the same genomic region of a single species (fig. 4 and table 2). Clades 1–4 had 65%, 23%, 75%, and 52% of the *RFLs* represented in these species-specific clusters, respectively. Considering only those species with whole-genome sequence information available, 68% of their *RFL* genes were present in 13 clusters comprising 0.13% of their combined genomes. For example, in rice, 50% of the identified *RFL*s were found within 320 kb of chromosome 10 (table 2).

**Table 2:** Details of the Species-Specific Genomic Regions Having a High Density of *RFL* Genes

Region of High RFL Density				No. of Genes Present in Cluster
Clade	Species	Genome Location (bp)	Size (kb)	
1	*Se. italica*	Ch8:29882484—31204264	1,322	5
1	*Z. mays*	Ch2:227716868—228633247	917	6
1	*Se. italica*	Ch7:15683154—15692828	9	2
2	*O. sativa*	Ch4:16684906—16757223	9	2
2	*H. vulgare*	Ch1:47176692—50263441	3,087	3
2	*B. distachyon*	Ch2:38479458—39012768	533	7
3	*A. thalians*	Ch1:4183066—4355929	172	4
3	*A. thalians*	Ch1:23176930—23988740	812	17
4	*S. bicolor*	Ch2:5169697—5744703	575	3
4	*Z. mays*	Ch8:76606724—76690742	84	2
4	*S. bicolor*	Ch5:2222303—2776884	554	9
4	*O. sativa*	Ch10:18823675—19143586	320	6
4	*O. sativa*	Ch8:374091—383986	10	2

Given the abundance of *RFL*s within these relatively small genome regions, these sites can be considered hotspots for *RFL* recombination that exhibit elevated rates of recombination relative to a neutral expectation. *RFL* genes within these clusters, at the same genome region, will be the youngest as they are still present within this *RFL* recombination hotspot. This implies that any list of candidate *PPR-Rf* genes can be further narrowed to *RFL*s present within these zones. These regions of active *RFL* generation contained known *Rf* genes, with the rice *Rf1* (Wang et al. 2006) and *Rf4* (Luo et al. 2013) genes being present in the *RFL*-rich region of rice chromosome 10. This allowed us to further refine the list of possible *Rf* genes in perennial ryegrass by looking for groups of tightly clustering sequences that show a similar pattern to other species. From Clade 2, three groups of perennial ryegrass *RFL*s meeting these criteria were identified, comprising of seven, eight, and five sequences, respectively (given in blue, fig. 4*B*). The first of these groups contained only sequences present in the OrthoMCL RFL cluster, the second group four sequences from this cluster and four from the original RFL genome scan and the third cluster four from the RFL cluster and one from the genome scan.

### Synteny Analysis

In order to identify the genome position of *RFL* generation in perennial ryegrass, a comparative genomics approach based on the Genome Zipper (Pfeifer et al. 2013) was applied. The *RFL*-rich zones from species with available genomic information were searched for conserved synteny with the genomes of other related plant species. The comparative genomics tool available at Ensemble Plants (http://plants.ensembl.org/index.html) was used to discern if any synteny exists between the *RFL*-rich genomic regions, from different species, within a single clade. This revealed no synteny between any *RFL*-rich regions both within each clade and between clades.

## Discussion

A bioinformatics pipeline targeting candidate *Rf* genes for CMS was successfully established and identified three clusters of possible *RFL* generation in perennial ryegrass. This pipeline, consisting of three complementing steps (supplementary fig. S1, Supplementary Material online), is based on genomic sequence data and thus can be used in any plant species for which such data are available. Validation of the pipeline in fully sequenced grass species such as rice, Brachypodium, and sorghum revealed that 50–90% of candidate *RFL* genes are found within no more than three genomic regions consisting of 0.1–0.01% of the genome. A similar approach could now be applied to cereals, where efficient access to *Rf* genes is an integral part of CMS-based hybrid production (Whitford et al. 2013).

The first step of this pipeline utilizes protein domain profile matrixes and sequence comparisons to identify PPR and RFL proteins from translated CDS. The second step involves orthologous clustering of multiple species, to identify *RFL* genes. This second step does not undermine the first step of this pipeline but is complimentary, as the first step identifies a more complete set of *RFL*s including pseudogenes and poorly annotated genes, both of which are important in identifying *RFL* recombination hotspots. The second step is also integral as it provides the data to complete the third and final step, which employs phylogenetic analysis to recognize areas of *RFL* diversification within the genome. This method not only identifies candidate *PPR-Rf* genes from restoring genotypes but also enables efficient identification of dynamic *RFL* clusters from nonrestoring phenotypes (Lurin et al. 2004; O'Toole et al. 2008; Li et al. 2012; Dahan and Mireau 2013).

### PPR and RFL Genes in Perennial Ryegrass

In the draft genome sequence of perennial ryegrass (Byrne et al. 2015), 373 *PPR* genes were identified and classified, revealing 25 *RFL*s. The number of *RFL*s identified here is consistent with other studies that have reported 10–30 *RFL*s per genome (Fujii et al. 2011), for example in Arabidopsis (Lurin et al. 2004). These *RFL*s have, on average, six more PPR domains than non-RFL PPR proteins. This possibly indicates that in perennial ryegrass, RFLs have a higher RNA sequence specificity than other PPR proteins. This was expected as known PPR-Rf proteins bind to a specific mRNA sequence whereas other PPRs have been shown to bind to multiple mRNAs (Zehrmann et al. 2009). Further evidence for multiple binding specificities comes from the number of transcript editing sites being present in mitochondrial genomes compared with PPRs with editing domains. The Arabidopsis mitochondrial genome encompasses 441 cytosine to uracil editing sites, although only 193 *PPR* genes, containing the E domain required for transcript editing, can be found in the nuclear genome (Giege and Brennicke 1999; Lurin et al. 2004). It appears that RFL proteins, unlike some other PPR proteins, are highly specialized, targeting a single transcript within the mitochondria (Barkan and Small 2014).

### Orthology-Based Strategies for RFL Identification

By using orthologous clustering, *RFL* genes from nine species were identified, showing that *RFL*s are distinct enough to be identified directly from whole-genome sequence data without first identifying the *PPR* gene family (Desloire et al. 2003; Geddy and Brown 2007; O'Toole et al. 2008). This was exemplified in figure 2*B* where the only cluster containing more than 50 *PPR*s was the *RFL* cluster. Strikingly, all known *Rf* genes that were present in the original genomes used for clustering were found in the *RFL* cluster. This also validates the sequence alignment and comparison approach used to identify *RFL*s from the whole set of *PPR* genes in perennial ryegrass. Non-*RFL PPR*s also clustered together with their orthologs from different species, but in contrast to *RFLs*, most of these clusters contained only one orthologous *PPR* gene per species.

Although the orthologous clustering and phylogenetic approach is an effective method to identify regions of active *RFL* generation, it was unable to identify all PPRs and was also less successful at identifying *RFL* genes, from perennial ryegrass, than the genome scanning approach. The effectiveness of the orthologous clustering and phylogenetic approach is dependent upon the type and quality of the input data. The type of data used is important as genomic sequence information may contain a more complete set of *RFL* genes than transcriptome data because of the tissue- and time-specific expression of *RFL* genes (Prasad et al. 2003). This is highlighted by the Italian ryegrass and meadow fescue transcriptomes, comprising relatively few *RFLs.* On the other hand, due to this tissue- and time-specific expression, transcriptome data could also be used to enrich for *Rf* genes by sampling from tissues known to be expressing *Rf* proteins, such as anthers (Kazama and Toriyama 2014). Although the use of genomic sequence data is preferable, individual *RFLs* can still be overlooked by orthologous clustering if they are poorly annotated or pseudogenes. Moreover, using incomplete genome assemblies as input data may not reveal all *RFL* clusters as they can be difficult to assemble, due to the repetitive features of *RFL-*rich genomic regions (Tsai et al. 2010). This was observed in barley, where an *RFL* was identified on an unordered contig from the same chromosome 6HS containing a recently mapped *Rf* locus that could not be associated with an *RFL* cluster (Ui et al. 2015). In cereals, further functional restorer loci have been described in wheat (Ma et al. 1995) and rye (*Secale cereal* L.) (Hackauf et al. 2012). The use of sequence data from restoring individuals, in conjunction with the pipeline described here could help to identify candidate *Rf-PPR* genes within the identified regions.

### Genome Regions of Active RFL Gene Generation

To identify the likely location of any *PPR-Rf* genes, a phylogenetic approach was applied to find clusters of highly similar *RFL* genes within single species, allowing the genomic regions of *RFL* generation to be distinguished. By comparing species with genome location information to perennial ryegrass, three regions of possible *RFL* generation were identified. Through further phylogenetic analysis of *RFL*s from several species, the fine structure of *RFL* organization in grasses was resolved and regions of novel *RFL* generation in species with positional genome information identified. This understanding of the architecture of *RFL* genes within other grass species led to the identification of similar groups of *RFL* genes in perennial ryegrass. Given the phylogenetic similarities between these groups, we can confidently assume that each of these groups of *RFL* genes in perennial ryegrass will be represented at single loci within the genome. These loci could be elucidated with more detailed genomic information or the use of a mapping population for genetic linkage mapping. Wild einkorn wheat, another species without genome location information, also showed a similar pattern with three tight clusters indicating the likelihood of three *RFL* generation loci.

The rate of recombination within the mitochondrial genome, which is the source of novel CMS mechanisms, is high (Kubo et al. 2011; Sloan et al. 2012), requiring a relatively rapid generation of new *RFL* genes through recombination driven diversifying selection (Fujii et al. 2011). The likelihood of functional *PPR-Rf* genes being present in these zones of active *RFL* generation is a function of how long it takes for fertility restoration to become fixed within a population (the time it takes for an *Rf* gene to restore CMS in an entire population) and the rate at which *RFL* genes are shuffled throughout the genome (how long a newly functional *Rf* gene is likely to stay within the genome region of active *RFL* generation). This suggests that if the rate of fixation is faster than the rate of shuffling, *Rf* genes will always be found within these *RFL* clusters. This is further borne out by the genome synteny results, showing a breakdown of synteny in the region of *RFL* generation zones, indicating that novel *RFL* generation occurs faster than speciation, unlike other *PPR* genes that are highly conserved between species. Similar findings were reported for barley and rye where *Rf* containing regions showed synteny to regions from rice, Brachypodium, and sorghum that contained no *RFLs* (Hackauf et al. 2012; Ui et al. 2015). These results indicate not only that *RFL*s are being shuffled around the genome at a rate faster than that of speciation but also that they are being rapidly lost when nonfunctional (Dahan and Mireau 2013).

In the four clades identified within the *RFL* cluster, all the dicot *RFL* genes fell within a single clade, representing the split between monocots and dicots. Although the dicot sequences were in a separate clade, the fact that *RFL*s from both monocot and dicot species were identified within a single cluster based on orthologous clustering is consistent with the hypothesis that monocot and dicot *RFL* genes share a common ancestor. This also suggests that this common ancestor is distinct from all other *PPR* genes and predates the monocot/dicot split, meaning that *RFL* genes evolved before this split (O'Toole et al. 2008).

### Accuracy and Usefulness of This Approach

The approach presented here allows efficient targeting of *RFL* containing genomic region(s) in multiple species. These regions have previously been shown to contain *Rf-PPR* genes (Bentolila et al. 2002; Kazama et al. 2008; Uyttewaal et al. 2008; Barr and Fishman 2010; Jo et al. 2010; Jordan et al. 2011; Kazama et al. 2014; Bisht et al. 2015) . In grasses, examples can be found in maize with the *Rf8* locus mapping to an *RFL* cluster on chromosome 2 (Meyer et al. 2011), and in rice with the *Rf1* (Wang et al. 2006) and *Rf4* (Luo et al. 2013) genes being present within the *RFL* cluster of rice chromosome 10. The most recent example is the *Rf6* restorer in rice (Huang et al. 2015) . *Rf6* was mapped to a 200 kb region on rice chromosome 8 which contains three *RFL*s identified in this study with one of these genes (Os08g01870) being located within 15 kb of the marker shown to be cosegregating with the restorer gene (Huang et al. 2012) . The only identified *PPR-Rf* gene that is located outside of the *RFL*-rich regions is *Rf1* from sorghum. The *Rf1* locus, most likely encoded by *PPR13*, is located as a single *PPR-Rf* gene on chromosome 8 although *PPR13* was not cloned from a restoring genotype (Klein et al. 2006). *PPR13* is different in its structure from all other identified *RFL-Rf* genes as it is of the PLS subtype and contains domains linked with RNA editing, indicating that the mechanism for restoration of the CMS phenotype may also be unique (Schmitz-Linneweber and Small 2008; Dahan and Mireau 2013). *PPR13* also exemplifies the complementarity of protein domain profile matrix scans and orthologous clustering, the latter of which would have been unable to detect a gene like *PPR13.*

The clustering approach assumes that newly functional *PPR-Rf* genes are the result of recombination events within an *RFL* genomic cluster and not an existing *RFL* that has gained a restoring function through the serendipitous recognition of a novel CMS causing transcript within the mitochondria. This balance will most likely differ between species and between populations of the same species under differing environmental conditions. It is important to note here that this approach will be most successful in identifying *PPR-Rf* genes in naturally occurring CMS systems (where the rapid evolution of *RFL*s has had time to overcome the damage in the mitochondria), but will also find traction in induced CMS systems where the CMS phenotype still has a mitochondrial ORF as its source and as such a possible *PPR-Rf* gene as a restorer.

### The Value of Rf Genes for CMS-Based Pollination Control in Forage Grasses

This pipeline provides an efficient first approach for *Rf* gene identification as it permits researchers to target the most likely genomic regions to contain *Rf* genes. Rapid identification of *Rf* candidate *RFL* genes will facilitate the development of functional markers for restoration of fertility, enabling efficient exploitation of CMS as a tool to control pollination for hybrid breeding in forage grasses. However, fertile hybrid seed is not necessarily needed for temporary forage production as biomass and not seed is the primary yield target (Islam et al. 2014). Indeed, it is often unwelcome as any partial or full restoration of male fertility during hybrid seed production would decrease the purity and value of that seed. Nevertheless, *Rf* gene identification is important to ensure that markers can be designed and populations screened to prevent unwanted fertility restoration. This will help to overcome the main challenge in outbreeding forage grasses with highly heterozygous genomes which is the maintenance of the CMS trait. The ability to rapidly identify individuals carrying an *Rf* gene within a breeding population would assist breeders in maintaining the commercially important CMS phenotype as well as ensuring hybrid seed purity. For breeding purposes, the exact position of the *Rf* gene does not need to be identified as genetic markers tightly linked to the functional *Rf* gene might be sufficient to identify restoring phenotypes. The approach used in this study can provide this by identifying *RFL* clusters within the genome allowing the relatively rapid identification of useful markers. Further dissection of *RFL* clusters, possible through BAC library screen and subsequent BAC clone sequencing, would allow the identification and cloning of the responsible *Rf* gene.

## Conclusion

Here, we have designed and implemented an in silico pipeline to identify candidate *Rf-PPR* genes and demonstrated its effectiveness by pinpointing known *Rf* genes. This study focused on perennial ryegrass and identified three regions of active *RFL* generation, providing excellent targets for marker development and future mapping approaches. Information is also provided for other species such as wild einkorn wheat, showing the wider applications of this method. As demonstrated, this pipeline can also be used to characterize *RFLs* in both monocots and dicots, to provide new insights into their evolution. The predictive power of this approach will improve as more genome sequence data becomes available. Knowledge of *RFL*-rich genomic regions within a genome might also be used for targeted sequencing of such regions in restorer plants and facilitate the expedient determination of *Rf* genes, the knowledge of which would not only be useful for breeding programs but also for fundamental research into nuclear/mitochondrial interactions.

## Supplementary Material

Supplementary Data
