# Supplementary material for: In Silico Identification of Candidate Genes for Fertility Restoration in Cytoplasmic Male Sterile Perennial Ryegrass (Lolium perenne L.)
Source: Genome Biol Evol. 2016 Mar 4;9(2):351–62. doi: 10.1093/gbe/evw047 (PMC5499803; doi:10.1093/gbe/evw047)
Supplement: Supplementary Data [file evw047_Supp.zip › Sup.docx]

**Sup. Fig. 1.** Work flow diagram illustrating the three-step bioinformatics pipeline used to isolate *RFL* genes in perennial ryegrass (*Lolium perenne* L.). Boxes with rounded corners contain bioinformatics processes, boxed with square corners contain input/output data.
