# Supplementary material for: In Silico Identification of Candidate Genes for Fertility Restoration in Cytoplasmic Male Sterile Perennial Ryegrass (Lolium perenne L.)
Source: Genome Biol Evol. 2016 Mar 4;9(2):351–62. doi: 10.1093/gbe/evw047 (PMC5499803; doi:10.1093/gbe/evw047)
Supplement: Supplementary Data [file evw047_Supp.zip › Supp. Fig. 1.pdf]

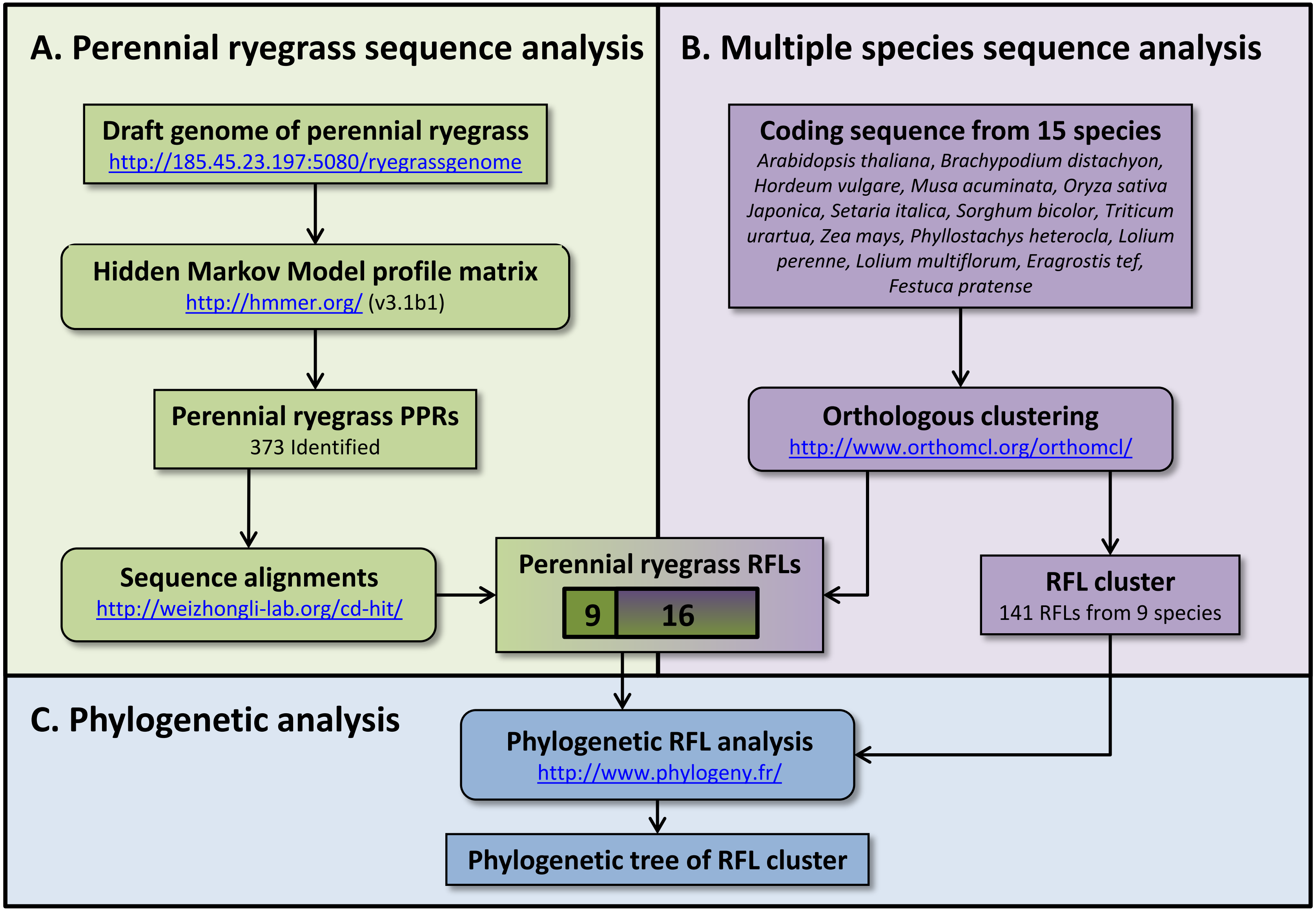
A. Perennial ryegrass sequence analysis
Draft genome of perennial ryegrass
http://185.45.23.197:5080/ryegrassgenome
Hidden Markov Model profile matrix
http://hmmer.org/ (v3.1b1)
Perennial ryegrass PPRs
373 Identified
Sequence alignments
http://weizhongli-lab.org/cd-hit/
Perennial ryegrass RFLs
9
16
B. Multiple species sequence analysis
Coding sequence from 15 species
Arabidopsis thaliana, Brachypodium distachyon, Hordeum vulgare, Musa acuminata, Oryza sativa Japonica, Setaria italica, Sorghum bicolor, Triticum urartua, Zea mays, Phyllostachys heterocla, Lolium perenne, Lolium multiflorum, Eragrostis tef, Festuca pratense
Orthologous clustering
http://www.orthomcl.org/orthomcl/
RFL cluster
141 RFLs from 9 species
C. Phylogenetic analysis
Phylogenetic RFL analysis
http://www.phylogeny.fr/
Phylogenetic tree of RFL cluster
